# Excessive health behaviors in sports: links of orthorexia nervosa and exercise addiction with well-being, exercise activity in sports categories, and gender effects

**DOI:** 10.3389/fnut.2024.1494958

**Published:** 2024-12-03

**Authors:** Hanna Wachten, Ramona Wurst, Sarah Paganini, Jana Strahler

**Affiliations:** ^1^Sportpsychology, Department of Sport and Sport Science, Albert-Ludwigs-University Freiburg, Freiburg, Germany; ^2^Department of Psychotherapy and System Neuroscience, Justus Liebig University Giessen, Giessen, Germany; ^3^Department of Health Psychology and Applied Diagnostics, University of Wuppertal, Wuppertal, Germany

**Keywords:** orthorexia nervosa, exercise addiction, healthy orthorexia, healthy eating, health behaviors, behavioral addiction, disordered eating, sports

## Abstract

**Background and aims:**

Orthorexia nervosa (OrNe), the obsessive fixation on healthy eating, and exercise addiction (ExAdd) have been discussed as correlated excessive health behaviors with potential mental health implications. The role of gender-and sports-specific differences remains unclear. This study aimed to investigate the links of ExAdd, OrNe, and the non-pathological interest in healthy eating (healthy orthorexia; HeOr) with well-being and exercise activity in various sports, considering gender effects.

**Methods:**

Data from two cross-sectional online surveys were combined (*N* = 1,064, 73.5% women, age = 28.23 ± 11.09), measuring ExAdd (Exercise Addiction Inventory Revised), OrNe and HeOr (Teruel Orthorexia Scale), well-being (WHO-5 Well-Being Index), exercise activity and sports (Physical Activity, Exercise, and Sport Questionnaire).

**Results:**

Well-being correlated with OrNe among women (*r* = −0.291) and not meaningfully with ExAdd and HeOr among men. Gender differences were not significant, except for the higher correlation of ExAdd with exercise activity in resistance and fitness sports among men. Regression analyses revealed that exercise activity, especially in health, resistance and fitness sports, was linked to HeOr, but not to OrNe. ExAdd was associated with exercise activity in endurance, resistance and fitness, ball and team, antigravitation, technical, aesthetic sports, and martial arts.

**Discussion:**

Men’s well-being may slightly benefit from ExAdd and an interest in healthy eating, while obsessive healthy eating may reduce women’s well-being. Although exercise activity is associated with ExAdd in a wide variety of sports, it does not explain the link between ExAdd and OrNe. Preventive measures should consider gender-specific risks in excessive health behaviors.

## 1 Introduction

The significance of exercise and healthy eating in enhancing and sustaining both mental and physical well-being has been underscored (1, 2). Nevertheless, excessive focus on health behaviors may undermine their positive impacts. In this regard, orthorexia nervosa (OrNe) and exercise addiction (ExAdd) were discussed as potential mental health disorders (3, 4). Still, mental illness and mental health are two distinct but related dimensions. According to the two continua model of mental health and illness, behaviors do not necessarily have to be classified as mental disorders to impact mental health (5, 6).

OrNe describes the obsessive preoccupation with self-imposed rigid dietary rules based on exaggerated perceptions of the health benefits and harms associated with food (7, 8). Various factors seem to contribute to the development and maintenance of OrNe, including perfectionism, health controllability and illness anxiety (9, 10). Although OrNe may lead to malnutrition, weight loss, distress, and impairment in psychosocial areas of functioning, its clinical relevance lacks sufficient evidence (3, 11). Assessment of OrNe’s clinical impact has been impeded by invalid screening methods (12, 13). Established instruments either neglected or solely measured the non-pathologic interest in healthy eating without addressing its pathological aspects (14, 15). Therefore, a two-dimensional model (Teruel Orthorexia Scale—TOS; 16) distinguishes between healthy orthorexia (HeOr) and OrNe, each associated with impairment and distress differently (16–18). OrNe’s and HeOr’s associations with well-being differ by gender (18, 19), highlighting the ambiguous interplay between (obsessive) healthy eating and mental health.

Besides interest in healthy eating, exercise may also manifest unhealthy dimensions. ExAdd denotes a potential behavioral addiction characterized by a high salience of exercise in the daily routines of affected individuals, accompanied by withdrawal symptoms and diminished control over frequency, intensity and/or duration of exercise sessions. The subordination of other activities and high training volumes lead to intrapsychic, social and occupational conflicts (20, 21). Contrary to the beneficial effects of exercise (22, 23), ExAdd is associated with psychopathological symptoms (24, 25). However, the impact of ExAdd on mental health and whether it outweighs the positive effects of exercise remains inconsistent and seems potentially moderated by gender. For example, two studies yielded inconsistent findings when they compared male and female cyclists at risk and not at risk for ExAdd regarding their mental quality of life with inactive participants (26, 27). Furthermore, the risk for ExAdd could vary by sports category performed, with tendentially higher risks in strenuous activities like endurance sports (28). However, exercise addiction research has mostly focused on athletes in endurance sports to date (21) and findings on sports categories at risk for ExAdd are sparse and heterogonous (4, 28).

Given the presumably shared motives of health optimization and maintenance, excessive exercise was discussed as a possible symptom of OrNe (29) and athletes have been considered at-risk populations for OrNe (30). As suggested by a prior meta-analysis (31), however, exercise seems to be of minor importance in OrNe (*r* = 0.12). Rather, both excessive health behaviors, OrNe and ExAdd, were moderately correlated (*r* = 0.29). Various factors may underly this association. Athletes in some sports prioritize healthy eating as a means to optimize performance or overall health (32). This may not only face elevated risks of eating disorders, e.g., in aesthetics, weight-class, endurance, resistance or antigravitation sports (33, 34), but also OrNe and ExAdd. Conclusions regarding the sport category-specific risk patterns are limited by methodological constraints and lack of differentiation between healthy and pathological orthorexic behavior and sports categories (31).

Examining the relationships between specific sports categories and both excessive health behaviors could enrich comprehension of these phenomena, shedding light on their interrelatedness and the motivating factors driving them (35, 36). Gender differences could further deepen the understanding of their association for several reasons. Firstly, manifestations of OrNe and ExAdd, as well as their impacts on mental health, may vary between genders (36). Secondly, gender-stereotypes regarding preferred sports may influence men’s and women’s participation interests (37). Lastly, men often engage in weight and resistance sports and eat less restrictive compared to women when experiencing disordered eating (38). The aim of this study was thus to expand knowledge of the relationship of OrNe, HeOr and ExAdd with well-being and exercise activity, considering sports categories and gender, with potential implications for the development of effective prevention and treatment strategies.

Based on the proposed clinical relevance of excessive health behaviors, we expected well-being to positively correlate with HeOr, but to correlate negatively with OrNe and ExAdd. These negative correlations are expected to be more pronounced in women (18, 26, 27). While we expected correlations between exercise activity and OrNe to be generally small (31), we explored whether OrNe and ExAdd show meaningful associations with exercise activity in the same sports categories.

## 2 Materials and methods

### 2.1 Participants and data collection

Data from two cross-sectional online surveys (conducted via www.soscisurvey.de) were combined. The studies differed in recruitment period (study 1: August to October 2019; study 2: April to June 2021) and advertisement using convenience sampling strategies to target general population (study 1: association of health with exercise; study 2: with eating behavior). University mailing lists and social media were used for recruitment, and flyers were displayed in public places, sports clubs, and gyms. Inclusion criteria were the minimum age of 18 and the self-assignment to male or female gender. Fifteen participants did not meet the criteria (*N* = 3 of diverse gender and *N* = 12 under the age of 18). The final sample comprised 1,064 participants (Study 1: *N* = 387; Study 2: *N* = 677).

### 2.2 Measures

Participants provided sociodemographic data including gender, age, body mass index (BMI), highest educational level within the German school system (none or lower secondary school, middle school, comprehensive school, matriculation examination), and eating style (vegan, lacto-vegetarian, ovo-lacto-vegetarian, ovo-lacto-vegetarian + fish and poultry, meat 1–2 times per month, omnivore).

The Teruel Orthorexia Scale (TOS; 16) was used to measure orthorexic eating behavior on the two subscales healthy orthorexia (HeOr; 9 items) and orthorexia nervosa (OrNe; 8 items). The items are rated on 4-point Likert scales ranging from “0—*completely disagree*” to “3—*completely agree*.” Higher sum scores indicate higher levels of OrNe (range: 0 to 24) and HeOr (range: 0 to 27), respectively. Cut-offs are not provided. Total samples’ internal consistencies were Cronbach’s *α* = 0.891 (sample 1: α = 0.966; sample 2: α = 0.891) for OrNe and Cronbach’s α = 0.905 (sample 1: α = 0.789; sample 2: α = 0.905) for HeOr.

The Revised Exercise Addiction Inventory [EAI-R; (39)] was employed to assess exercise addiction. The six items are rated on 6-point Likert scales ranging from “1—*strongly disagree*” to “6—*strongly agree*.” Adapting the cut-offs of the unrevised version (40), EAI-R sum scores (range: 6 to 36) ≤ 14 indicated the absence of symptoms, 15 to 28 indicated the presence of symptoms, and ≥ 29 indicated a risk for ExAdd. Internal consistency was Cronbach’s *α* = 0.924 for the total sample (sample 1: α = 0.879, sample 2: α = 0.924).

Well-being was measured by the WHO-5 Well-Being Index [WHO-5, (41)]. The 5 items are rated on 6-point Likert-scales ranging from “0—*none of the time*” to “5—*all of the time*.” The scores are summed up and multiplied by 4 to compute the percentage scale of well-being (range: 0 to 100). The WHO-5 is a well-established and validated tool (42). Internal consistency was Cronbach’s *α* = 0.915 for the total sample (sample 1: α = 0.737 sample 2: α = 0.914).

Exercise activity (minutes per week) and sports categories were measured by the sport and exercise activity subscale of the Physical Activity, Exercise, and Sport Questionnaire {[Bewegungs-und Sportaktivität Fragebogen] BSA-F; (43)}. Although validated, the BSA’s reliability has not been estimated yet. Participants indicate up to three sports they engaged in over the past 4 weeks. For each indicated sport, participants report the frequency of exercise sessions and their typical duration in minutes. Using an expert based approach before data collection, all authors classified exercise activities into sports categories based on those used in eating disorder research (44, 45). Ambiguities were resolved through discussion until full agreement was reached, resulting in the following nine sports categories: Endurance sports (e.g., jogging), resistance and fitness sports (e.g., weight training), health sports (e.g., yoga), ball and team sports (e.g., soccer), antigravitation sports (e.g., climbing), technical sports (e.g., surfing), aesthetic sports (e.g., dancing), rehabilitation sports (e.g., back strengthening), and martial arts (e.g., boxing). Subsequently, the categorization of self-reported sports and computation of exercise activity in each sports category (minutes per week) was performed using the fitevalapp, which was developed for the computation of exercise activity scales (46).

### 2.3 Statistical analyses

All analyses were carried out using R [4.2.0, (47)]. We used Welch-tests to compare women and men with regard to age, BMI, EAI-R, HeOr, OrNe, WHO-5, and BSA-F (48, 49). For categorial data (gender, educational level, eating style and EAI-R risks), we computed Fisher’s exact tests. Since participants could indicate up to three sports categories, direct comparison of sports categories was not feasible. Instead, we conducted exploratory 2×2 ANOVAs with independent variables sports (no sports vs. indicated sports category), gender (women vs. men) and dependent variables (EAI-R; TOS HeOr; TOS OrNe; WHO-5) for each of the nine sports categories. To address heteroscedasticity and unbalanced sample sizes, we used a generalized *p*-value approach with parametric bootstrapping (50, 51) of the *twowaytests* package (52). Due to bivariate non-normality [*mvnormalTest* package, (53, 54)], we computed studentized tests with 9,999 permutations to test Pearson correlations of EAI-R, TOS HeOr, and TOS OrNe with WHO-5 and BSA-F (exercise activity in total and per sports category) for the total sample and separately for men and women (55, 56). We used the *nptest* package (57) and considered only *r* ≥ 0.200 as meaningful (58). Subsequently, we tested the equality of the four pairs of significant and meaningful gender-specific correlations using Fisher’s z-tests. To examine the relative importance of sports, we computed three regression analyses with all nine sports categories explaining the three outcomes EAI-R, TOS HeOr, and TOS OrNe. We checked for influential values and multicollinearity using Cook’s Distance < 0.5 and VIF < 5, respectively. One influential value was excluded in the TOS HeOr regression model. Due to non-normality and heteroscedasticity of residuals, we used HC4 robust standard errors (59).

## 3 Results

### 3.1 Sample characteristics

**Figure 1:**
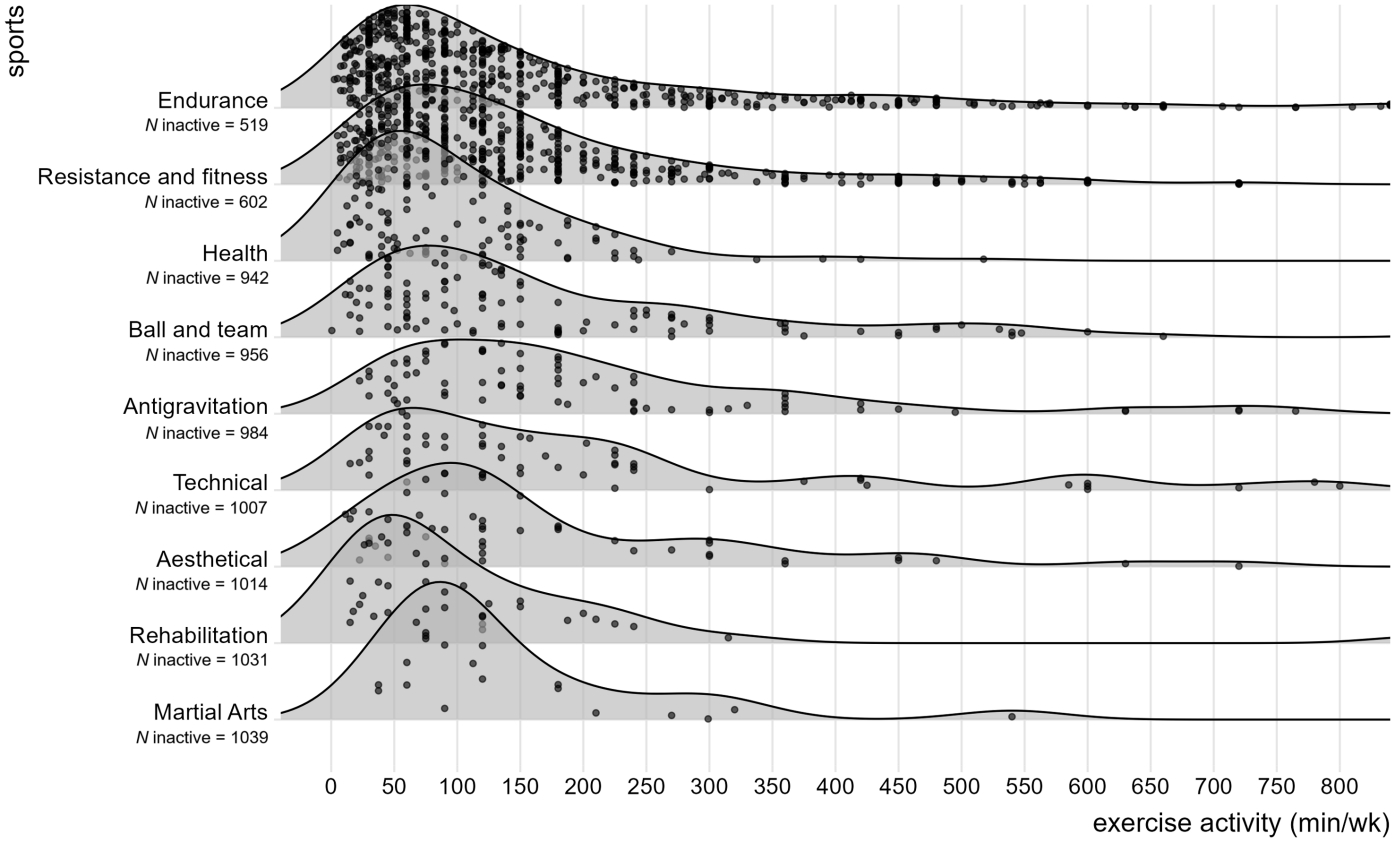
Ridgeline plot of exercise activity separated by sports categories among the active sample (*N* = 844), with each point representing an individual value.

Overall, the total sample was mainly female, about half of the participants reported no dietary restrictions and the majority was highly educated. Men were older [*t*(376.58) = 6.695, *p* < 0.001], of higher BMI [*t*(545.58) = 10 0.436, *p* = 0.040], less likely to restrict their diet (*p* = 0.003), reported to be more active [*t*(436.85) = 4.240, *p* < 0.001] and of higher well-being [*t*(544.32) = 5.526, *p* < 0.001] compared to women. Regarding excessive health behaviors, men showed higher EAI-R scores [*t*(493.02) = 2.871, *p* = 0.004] and women higher TOS OrNe scores [*t*(734.97) = −4.213, *p* < 0.001]. Men and women did not differ in TOS HeOr scores [*t*(464.14) = −1.533, *p* = 0.126] and frequencies of EAI-R risk categories (*p* = 0.120), but in educational levels (*p* = 0.003). Within the total sample, 20.7% of participants (*N* = 220) were inactive, i.e., reported no exercise activity within the last 4 weeks. Of all active participants (*N* = 844), 39.9% reported to engage in sports within one category (*N* = 337), 44.4% of two categories (*N* = 375) and 15.6% in the maximum of three categories (*N* = 132). For further details see Figure 1 and Table 1.

**Table 1:** Descriptive statistics *M* ± *SD* for metric variables and *N* (%) for categorial variables of the total sample (*N* = 1,064), women (*N* = 783) and men (*N* = 281).

	Total sample	Women	Men
Age	28.23 ± 11.09	26.65 ± 9.42	32.64 ± 13.88
BMI	22.62 ± 3.81	21.95 ± 3.75	24.47 ± 3.37
Educational level			
None or lower secondary school	7 (0.7)	5 (0.6)	2 (0.7)
Middle school	68 (6.4)	52 (6.6)	16 (5.7)
Comprehensive school	27 (2.5)	11 (1.4)	16 (5.7)
Matriculation examination	962 (90.4)	715 (91.3)	247 (87.9)
Eating style			
Vegan	118 (11.1)	98 (12.5)	20 (7.1)
Lacto-vegetarian	15 (1.4)	14 (1.8)	1 (0.4)
Ovo-lacto-vegetarian	214 (20.1)	191 (24.4)	23 (8.2)
Ovo-lacto-vegetarian + fish & poultry	22 (2.1)	22 (2.8)	-
Meat 1–2 times per month	133 (12.5)	107 (13.7)	26 (9.3)
Omnivore	552 (51.9)	346 (44.2)	206 (73.3)
EAI-R	18.42 ± 6.99	18.06 ± 6.96	19.45 ± 6.98
Asymptomatic	300 (28.2)	234 (29.9)	66 (23.5)
Symptomatic	683 (64.2)	491 (62.7)	192 (68.3)
At risk	81 (7.6)	58 (7.4)	23 (8.2)
TOS OrNe	3.71 ± 4.57	4.00 ± 4.92	2.89 ± 3.31
TOS HeOr	12.62 ± 5.25	12.78 ± 5.14	12.20 ± 5.54
BSA-F exercise activity	243.02 ± 263.74	221.17 ± 249.85	303.92 ± 290.97
WHO-5	54.88 ± 20.74	52.90 ± 21.02	60.40 ± 18.93

EAI-R, exercise addiction inventory revised; TOS HeOr, healthy orthorexia subscale of the Teruel Orthorexia Scale; TOS OrNe, orthorexia nervosa subscale of the Teruel Orthorexia Scale; BSA-F, Physical Activity, Exercise, and Sport Questionnaire;; WHO-5, WHO-5 well-being scale.

### 3.2 Comparison of active and inactive participants in excessive health behaviors and well-being

Among active participants, 64.1% engaged in endurance sports (*N* = 545), 54.9% in resistance and fitness sports (*N* = 463), 14.2% in health sports (*N* = 122), 12.8% in ball and team sports (*N* = 108), 9.3% in antigravitation sports (*N* = 80), 6.8% in technical sports (*N* = 57), 5.8% in aesthetic sports (*N* = 50), 3.8% in rehabilitation sports (*N* = 33), and 2.9% in martial arts (*N* = 25). Means and standard deviations of exercise activity (min/wk), OrNe, HeOr and WHO-5 separated by sports categories including ANOVA results are presented in Table 2. The exact *p*-values can be found in the supplements. All active participants in the various sports categories had significantly higher EAI-R scores than the group of inactive participants (main effects of sports participation *p* < 0.050). The ANOVAs revealed a significant interaction between gender and participation in resistance and fitness sports for EAI-R (*p* = 0.031). Specifically, women scored higher than men on the EAI-R among the inactive participants, whereas men had higher scores than women when they were participating in resistance and fitness sports. Compared to inactive participants, elevated TOS OrNe scores were associated with participation in resistance and fitness sports, rehabilitation sports, and martial arts (main effects *p* < 0.050), but not the other sport categories. Except for technical, aesthetic and rehabilitation sports, active participants showed significantly higher TOS HeOr scores than inactive participants (main effects of sports participation *p* < 0.05). A significant interaction effect emerged for gender and participation in health sports in HeOr (*p* = 0.038). Active participants had significantly higher WHO-5 scores than inactive participants, except for rehabilitation sports and martial arts (main effects of sports participation *p* < 0.05).

**Table 2:** Descriptive statistics *M* ± *SD* of exercise addiction, orthorexia nervosa, healthy orthorexia and well-being of inactive participants and those who engage in sports separated by gender including the generalized *p*-values of the main effects of the parametric bootstrapping 2×2 ANOVAs.

Sports categories	*N*	BSA-F	EAI-R	TOS OrNe	TOS HeOr	WHO-5
No sports						
Total	220	-	12.76 ± 6.57	3.27 ± 4.43	10.35 ± 5.38	46.33 ± 22.88
Women	167	-	12.81 ± 6.66	3.62 ± 4.80	10.65 ± 5.19	44.38 ± 23.51
Men	53	-	12.60 ± 6.35	2.15 ± 2.74	9.42 ± 5.89	52.45 ± 19.76
Endurance						
Total	545	182.04 ± 208.80	20.31 ± 6.31	3.82 ± 4.58	13.63 ± 5.09	58.89 ± 19.19
Women	385	159.89 ± 181.11	20.01 ± 6.40	4.08 ± 4.90	13.71 ± 4.95	56.87 ± 19.16
Men	160	235.35 ± 256.73	21.04 ± 6.05	3.21 ± 3.62	13.43 ± 5.43	63.75 ± 18.45
ANOVA			*p*_S_*** *p*_G_ *p*_SxG_	*p*_S_ *p*_G_** *p*_SxG_	*p*_S_*** *p*_G_ *p*_SxG_	*p*_S_*** *p*_G_*** *p*_SxG_
Resistance and fitness						
Total	463	159.13 ± 147.74	20.94 ± 5.95	3.91 ± 4.46	13.98 ± 4.98	56.86 ± 19.19
Women	339	139.62 ± 132.52	20.31 ± 5.90	4.16 ± 4.90	14.03 ± 4.90	54.91 ± 19.34
Men	124	212.47 ± 172.65	22.67 ± 5.75	3.22 ± 3.62	13.85 ± 5.22	62.03 ± 17.86
ANOVA			*p*_S_*** *p*_G_*** *p*_SxG_*	*p*_S_* *p*_G_** *p*_SxG_	*p*_S_*** *p*_G_ *p*_SxG_	*p*_S_*** *p*_G_*** *p*_SxG_
Health						
Total	122	110.87 ± 154.08	18.60 ± 5.58	3.48 ± 4.28	14.13 ± 4.64	57.12 ± 20.13
Women	111	111.54 ± 159.07	18.65 ± 5.65	3.66 ± 4.42	13.95 ± 4.71	56.94 ± 20.19
Men	11	104.09 ± 94.20	18.09 ± 4.99	1.73 ± 1.79	15.91 ± 3.48	58.91 ± 20.40
ANOVA			*p*_S_*** *p*_G_ *p*_SxG_	*p*_S_ *p*_G_*** *p*_SxG_	*p*_S_*** *p*_G_ *p*_SxG_*	*p*_S_*** *p*_G_ *p*_SxG_
Ball and team						
Total	108	184.68 ± 167.67	21.40 ± 5.75	3.56 ± 4.08	11.63 ± 4.64	58.48 ± 18.25
Women	58	174.83 ± 146.29	20.24 ± 5.75	4.00 ± 4.81	11.36 ± 4.15	54.00 ± 18.34
Men	50	196.11 ± 190.38	22.41 ± 5.52	3.06 ± 2.99	11.94 ± 5.17	63.68 ± 16.87
ANOVA			*p*_S_*** *p*_G_ *p*_SxG_	*p*_S_ *p*_G_* *p*_SxG_	*p*_S_* *p*_G_ *p*_SxG_	*p*_S_*** *p*_G_** *p*_SxG_
Antigravitation						
Total	80	280.56 ± 324.09	18.85 ± 6.36	3.48 ± 4.91	13.04 ± 5.28	59.65 ± 19.35
Women	53	328.63 ± 372.03	18.85 ± 6.73	4.51 ± 5.63	13.62 ± 5.39	57.21 ± 19.72
Men	27	186.20 ± 168.43	18.85 ± 5.70	1.44 ± 1.91	11.89 ± 4.96	64.44 ± 18.00
ANOVA			*p*_S_*** *p*_G_ *p*_SxG_	*p*_S_ *p*_G_*** *p*_SxG_	*p*_S_*** *p*_G_ *p*_SxG_	*p*_S_*** *p*_G_* *p*_SxG_
Technical						
Total	57	240.76 ± 253.05	19.44 ± 5.46	3.02 ± 3.87	11.61 ± 4.46	60.63 ± 19.28
Women	46	242.33 ± 264.93	19.20 ± 5.41	3.04 ± 4.17	11.50 ± 4.46	59.22 ± 20.02
Men	11	234.20 ± 206.67	20.45 ± 5.82	2.91 ± 2.34	12.09 ± 4.68	66.55 ± 15.21
ANOVA			*p*_S_*** *p*_G_ *p*_SxG_	*p*_S_ *p*_G_* *p*_SxG_	*p*_S_ *p*_G_ *p*_SxG_	*p*_S_*** *p*_G_* *p*_SxG_
Aesthetic						
Total	50	172.88 ± 158.63	19.90 ± 5.46	3.86 ± 4.90	11.64 ± 4.86	57.52 ± 17.08
Women	47	174.02 ± 163.16	19.74 ± 5.26	3.91 ± 5.04	11.98 ± 4.77	57.79 ± 16.84
Men	3	155.00 ± 60.62	22.33 ± 9.29	3.00 ± 2.00	6.33 ± 3.06	53.33 ± 24.44
ANOVA			*p*_S_** *p*_G_ *p*_SxG_	*p*_S_ *p*_G_* *p*_SxG_	*p*_S_ *p*_G_* *p*_SxG_	*p*_S_* *p*_G_ *p*_SxG_
Rehabilitation						
Total	33	117.80 ± 155.42	15.97 ± 5.97	3.45 ± 4.40	11.85 ± 6.31	47.64 ± 21.72
Women	27	110.46 ± 165.37	16.56 ± 6.20	4.07 ± 4.63	13.04 ± 5.98	47.11 ± 21.21
Men	6	150.83 ± 103.83	13.33 ± 4.27	0.67 ± 1.03	6.50 ± 5.13	50.00 ± 25.89
ANOVA			*p*_S_* *p*_G_ *p*_SxG_	*p*_S_* *p*_G_*** *p*_SxG_	*p*_S_ *p*_G_* *p*_SxG_	*p*_S_ *p*_G_ *p*_SxG_
Martial Arts						
Total	25	140.56 ± 113.83	24.16 ± 6.50	6.60 ± 6.46	14.84 ± 5.89	56.00 ± 18.87
Women	13	156.73 ± 140.55	23.92 ± 5.6	5.23 ± 5.86	14.31 ± 5.66	50.46 ± 18.51
Men	12	123.04 ± 77.96	24.42 ± 7.63	8.08 ± 6.99	15.42 ± 6.32	62.00 ± 18.09
ANOVA			*p*_S_*** *p*_G_ *p*_SxG_	*p*_S_* *p*_G_* *p*_SxG_	*p*_S_** *p*_G_ *p*_SxG_	*p*_S_ *p*_G_* *p*_SxG_

EAI-R, exercise addiction inventory revised; TOS HeOr, healthy orthorexia subscale of the Teruel Orthorexia Scale; TOS OrNe, orthorexia nervosa subscale of the Teruel Orthorexia Scale; BSA-F, exercise activity (min/wk) of the Physical Activity, Exercise, and Sport Questionnaire; WHO-5, WHO-5 well-being scale. *p*_S_, significance of the main effect of sports (sports vs. no sports); *p*_G_, significance of the main effect of gender (women vs. men); *p*_SxG_, significance of the interaction effect of sports and gender (***p < 0.001, **p < 0.010, *p < 0.050).

### 3.3 Associations of excessive health behaviors with well-being and exercise activity

The EAI-R was significantly correlated with both orthorexia subscales (TOS OrNe, *r* = 0.346, *t* = 8.777, *p* < 0.001, and HeOr, *r* = 0.369, *t* = 10.629, *p* < 0.001), which also correlated significantly (*r* = 0.440, *t* = 10.916, *p* < 0.001). The Pearson correlations of EAI-R, TOS OrNe, and TOS HeOr with WHO-5 and BSA-F exercise activity in general as well as for each sports category are displayed in Table 3. The WHO-5 was negatively correlated with TOS OrNe solely within the total and female sample. Among both men and women, the EAI-R was positively and meaningfully associated with BSA-F exercise activity in total, and with exercise activity in endurance sports, as well as resistance and fitness sports. BSA-F exercise activity in resistance and fitness sports was correlated in both genders with TOS HeOr as well. Gender differences were significant for the correlation between EAI-R and exercise activity in resistance and fitness sports, *z* = −2.011, *p* = 0.044, with higher correlations in men. Neither the correlation of EAI-R with total exercise activity, *z* = −263, *p* = 0.207, with endurance exercise activity, *z* = 1.172, *p* = 0.241, nor the correlation of TOS HeOr with exercise activity in resistance and fitness sports, *z* = −0.893, *p* = 0.372, differed significantly between men and women.

**Table 3:** Pearson correlations^a^ of the total sample (*N* = 1,064) and separately for women (*N* = 783) and men (*N* = 281).

	EAI-R			TOS OrNe			TOS HeOr		
	Total	Women	Men	Total	Women	Men	Total	Women	Men
WHO-5	0.050	−0.005	0.165**	−0.269***	−0.291***	−0.108	0.091**	0.067	0.198**
BSA-F	0.481***	0.456***	0.523***	0.061	0.089	0.042	0.207***	0.165***	0.219***
Endurance	0.281***	0.305***	0.229***	0.012	0.037	0.005	0.109***	0.117***	0.120**
Resistance and fitness	0.371***	0.329*** ^**b**^	0.448*** ^**b**^	0.051	0.051	0.118	0.246***	0.235***	0.293***
Health	−0.008	−0.001	−0.007	−0.012	−0.021	−0.029	0.069***	0.064**	0.104*
Ball and team	0.155***	0.132**	0.182***	0.013	0.056	−0.030	−0.056*	−0.025	−0.090
Antigravitation	0.067	0.070	0.068	0.059	0.080	−0.099**	0.062	0.071	−0.032
Technical	0.053*	0.055*	0.058	−0.025	−0.032	−0.003	−0.040	−0.044	−0.035
Aesthetic	0.077*	0.098*	0.027	0.020	0.014	−0.010	−0.015	−0.011	−0.102
Rehabilitation	0.019	0.044	−0.072***	−0.017	−0.004	−0.101*	−0.023	−0.004	−0.095
Material Arts	0.122**	0.116*	0.124*	0.075	0.038	0.246*	0.033	0.024	0.063

EAI-R, exercise addiction inventory revised; TOS HeOr, healthy orthorexia subscale of the Teruel Orthorexia Scale; TOS OrNe, orthorexia nervosa subscale of the Teruel Orthorexia Scale; BSA-F, exercise activity (min/wk) of the Physical Activity, Exercise, and Sport Questionnaire; WHO-5, WHO-5 well–being scale. Bold values indicate meaningful correlations r ≥ 0.200. *** *p* < 0.001 ** *p* < 0.01 * *p* < 0.05.

a Tested using studentized tests with 9,999 permutations.

b Gender difference according to Fisher’s *z*-test at *p* < 0.05.

When controlling for the variance explained by other sports categories, the EAI-R was significantly associated with BSA-F exercise activity in martial arts (*b* = 0.031, *t* = 2.901, *p* = 0.004), resistance and fitness sports (*b* = 0.021, *t* = 13.455, *p* < 0.001), ball and team sports (*b* = 0.016, *t* = 6.914, *p* < 0.001), aesthetic sports (*b* = 0.014, *t* = 4.195, *p* < 0.001), endurance sports (*b* = 0.013, *t* = 7.494, *p* < 0.001), technical sports (*b* = 0.007, *t* = 3.213, *p* = 0.001), and antigravitation sports (*b* = 0.006, *t* = 2.015, *p* = 0.044) in descending order, *R^2^* = 0.293 (adjusted *R^2^* = 0.287), *F*(9, 1,054), = 48.583, *p* < 0.001. TOS OrNe was not significantly explained by exercise activities in any sports categories, *R*^2^ = 0.014 (adjusted *R^2^* = 0.005), *F*(9, 1,054), = 1.635, *p* = 0.101, but TOS HeOr was, *R^2^* = 0.100 (adjusted *R^2^* = 0.092), *F*(9, 1,053), = 12.964, *p* < 0.001. BSA-F exercise activity in health sports (*b* = 0.014, *t* = 4.495, *p* < 0.001), resistance and fitness sports (*b* = 0.011, *t* = 8.381, *p* < 0.001), endurance sports (*b* = 0.004, *t* = 3.849, *p* < 0.001), and antigravitation sports (*b* = 0.003, *t* = 1.968, *p* = 0.049) significantly predicted TOS HeOr. Regression coefficients of all three models are presented in Table 4.

**Table 4:** Coefficients of regression models explaining exercise addiction, orthorexia nervosa, and healthy orthorexia.

BSA-F	EAI-R		TOS OrNe		TOS HeOr	
	*b*	95% CI	*b*	95% CI	*b*	95% CI
Endurance	0.013***	[0.009, 0.016]	0.000	[−0.001, 0.002]	0.004***	[0.002, 0.005]
Resistance and fitness	0.021***	[0.018, 0.025]	0.002	[−0.001, 0.004]	0.011***	[0.008, 0.014]
Health	0.003	[−0.017, 0.023]	−0.001	[−0.009, 0.008]	0.014***	[0.008, 0.020]
Ball and team	0.016***	[0.011, 0.021]	0.001	[−0.004, 0.006]	−0.003	[−0.007, 0.000]
Antigravitation	0.006*	[0.000, 0.012]	0.003	[−0.003, 0.008]	0.003*	[0.000, 0.007]
Technical	0.007**	[0.003, 0.012]	−0.001	[−0.004, 0.001]	−0.002	[−0.006, 0.002]
Aesthetic	0.014***	[0.007, 0.020]	0.002	[−0.008, 0.012]	−0.001	[−0.011, 0.009]
Rehabilitation	0.001	[−0.016, 0.018]	−0.003	[−0.017, 0.012]	−0.006	[−0.025, 0.013]
Material Arts	0.031**	[0.010, 0.051]	0.012	[−0.000, 0.025]	0.005	[−0.004, 0.014]

EAI-R, exercise addiction inventory revised; TOS HeOr, healthy orthorexia subscale of the Teruel Orthorexia Scale; TOS OrNe, orthorexia nervosa subscale of the Teruel Orthorexia Scale; BSA-F, exercise activity (min/wk) of the Physical Activity, Exercise, and Sport Questionnaire (***p < 0.001 **p < 0.01 *p < 0.05).

## 4 Discussion

This study investigated the relationship of HeOr and the excessive health behaviors OrNe and ExAdd with well-being and exercise activity focusing on sports categories and gender effects. We found a positive but negligible correlation between HeOr and well-being. Only partly aligning with our hypothesis, well-being was negatively associated with OrNe, but not correlated with ExAdd. Stratifying by gender, we observed a positive, but not meaningful small correlation between HeOr and well-being in men only, while a negative, small to moderate correlation with OrNe was evident in women but not in men. As expected, OrNe was not meaningfully associated with weekly exercise activity, except for martial arts among men (12 men, 13 women). Regression analysis supported this finding by showing no link between OrNe and exercise activity in any sports category. In contrast, HeOr was associated with exercise activity in health, resistance and fitness, endurance, and antigravitation sports. As expected, ExAdd was moderately to strongly correlated with weekly exercise activity in total. Stratifying by sports category, meaningful small to moderate links only emerged for resistance and fitness sports and endurance sports. Men exhibited a significantly higher correlation with fitness and resistance sports than women. Overall, exercise activity separated by sports category significantly explained a high proportion of the variance in ExAdd. Martial arts, resistance and fitness sports, ball and team sports, aesthetic sports, endurance sports, technical sports, and antigravitation sports contributed to the regression model in descending order.

### 4.1 Links of excessive health behaviors with well-being in men and women

The negative correlation between well-being and obsessive preoccupation with healthy eating was mainly driven by the female sample, while well-being was positively associated with interest in healthy eating in men. These gender-specific associations suggest that women do not generally benefit from a non-pathological interest in healthy eating in terms of their psychological well-being, but rather experience decreased well-being when fixating obsessively on healthy diet. Conversely, men’s well-being increased with the interest in healthy eating and was unrelated to the obsessive fixation on dietary behaviors. Women tend to hold stronger beliefs about the benefits of healthy eating and are more likely to restrict their diet accordingly (60, 61), but may not receive the expected mental health benefits. These results align with previous gender-moderated associations of well-being, showing a negative link with OrNe in women and a positive link with HeOr in men (18). Stutts (17) did not differentiate between OrNe and HeOr and observed a positive relationship between OrNe and well-being in men. Overall, OrNe appears to be associated with decreased mental health in women only.

Unexpectedly, men’s well-being slightly increased with higher ExAdd scores. To our knowledge, this is the first study indicating a positive link between ExAdd and well-being in men. Previous studies either failed to find a correlation between ExAdd and measurements of mental health (26) or found a negative link (25). Female cyclists at risk for ExAdd had the same mental quality of life as inactive women, while men might benefit from exercise regardless of their ExAdd risk (26, 27). Within our sample, active participants, except those who engaged in rehabilitation sports, showed higher well-being than inactive participants as well. In summary, ExAdd may not inherently negate the favorable impacts of exercise on mental health.

### 4.2 Sports categories at risk for excessive health behaviors in men and women

The links of exercise activity with obsessive and non-pathological interests in healthy eating varied across sports. Specifically, only martial arts exercise activity was associated with OrNe among men. Due to the insufficient sample size of men reporting martial arts participation, the correlation must be interpreted cautiously and highlights an interesting research gap. Given their high OrNe mean scores compared to inactive participants, male martial artists may be obsessively focused on nutrition, combining body mass manipulation with fueling their bodies for performance (62). HeOr was associated with health, resistance and fitness, endurance, and antigravitation sports. In line, previous findings found higher associations with exercise or increased prevalence of OrNe in athlete populations when using non-pathological measures for OrNe, such as the ORTO-15 (30, 63, 64). Extending the findings of a former meta-analysis (31), our results confirm that the obsessive fixation on healthy eating is unrelated to exercise activity across sports.

Bivariate correlations pointed to the strongest links of ExAdd with resistance and fitness sports, especially in men, and endurance sports in the total sample. While there is a long tradition of research on ExAdd in endurance sports [e.g., (65)], less attention has been given to resistance and fitness sports, which were frequently practiced in our sample. Surprisingly, our results indicated even stronger links between ExAdd and resistance and fitness sports than endurance sports, since their confidence intervals of regression slope coefficients did not overlap. Resistance and fitness sports are practiced due to various reasons and motives such as improvement of health, appearance and affect regulation (66). Strict training schedules, external motivation (such as seeking social recognition), social media use, disordered eating practices, younger age or obsessive passion could contribute to the development of ExAdd of athletes in resistance and fitness sports (67–69). Moreover, the gender effect suggests that men may use resistance and fitness sports in the context of disordered eating behavior to increase muscularity (38, 68). However, ExAdd was not exclusively associated with body weight and composition-dependent sports (33, 45).

Indeed, ExAdd showed links with exercise activity in ball and team, and technical sports. Due to the inflexible nature of training in team sports, athletes affected by ExAdd are considered “lone wolves,” who prefer practicing individual sports, either instead of or in addition to team sports (70). Our findings challenge this assumption, since participation in ball and team sports was associated with ExAdd, even when controlling for other sports categories. Consequently, elevated risks for ExAdd cannot be fully attributed to additionally practiced exercise activity in individual sports. Athletes at risk for ExAdd may perceive high social obligations to teammates, leading to negative emotional consequences when missing training opportunities or showing low individual performances (71). In line with this interpretation, two studies found no differences between team and individual sports in their risks for ExAdd (72, 73). All in all, our results shed light on the wide range of sports associated with ExAdd, with only health and rehabilitation sports being unrelated.

Beyond sport type, the competitional sports level also seems to play a significant role in ExAdd. While studies report high prevalence rates of ExAdd among elite athletes, such as over 40% among competitive triathletes (74), it has been suggested that elite athletes may interpret self-report measures differently than recreational athletes. For elite athletes, high ExAdd scores may reflect strong professional commitment rather than addiction, as their extensive training typically aligns with their career goals and does not necessarily lead to impairment (75). Nonetheless, evidence suggests that disregarding medical advice to cease exercise—likely due to withdrawal symptoms—is associated with ExAdd risks, regardless of the sports level (76). In light of the positive link of ExAdd and well-being within our sample, withdrawal symptoms and distress faced by athletes at risk of ExAdd should be further investigated.

### 4.3 Strengths and limitations

The present study encompasses both strengths and limitations. Notably, the merged samples offer a high sample size and variance in weekly exercise activity across various sports. Participants’ engagement in multiple sports likely mirrors typical recreational athlete behavior, since the majority of participants engaged in more than one sports category. Participants’ engagement in multiple sports likely mirrors typical recreational athlete behavior, since the majority of participants engaged in more than one sports category. Our dimensional approach enables the examination of unique associations with exercise activity in specific sports categories while controlling for others, adding value beyond direct group comparisons of sports categories. However, reliance on self-reported exercise activity and convenience sampling may have introduced biases, such as self-selection and social desirability, which limit the validity of our results (77). Although recruitment took place in different locations, we cannot completely exclude overlap of participants across samples. The unequal gender distribution and small sample size of participants, who practiced martial arts (*N* = 25) and rehabilitation sports (*N* = 33), constrain generalizability. The cross-sectional design precludes causal conclusions, such as whether increased sports participation raises ExAdd risks or if exercise activity increases due to ExAdd. Furthermore, without measuring disordered eating, we could not differentiate between instrumental exercise aimed at improving appearance and ExAdd (4). Prospective data are needed to unravel the etiology of excessive health behaviors in men and women.

## 5 Conclusion

Although pathological orthorexic eating and exercise addiction may co-occur and correlate moderately (31), their link can neither be explained by restrictions in well-being nor participation in specific sports categories. A non-pathological interest in healthy eating and ExAdd may slightly enhance well-being in men, but OrNe may be detrimental to women’s well-being only. Men and women may differ in how they experience and are mentally affected by these (excessive) health behaviors. Our findings highlight the importance of distinguishing between HeOr and OrNe (78). Exercise activity, especially in health, resistance and fitness sports, may correlate with a non-pathological interest in healthy eating, but not with OrNe. While athletes may not be particularly prone to obsess over healthy eating, they may be susceptible to ExAdd in a broad range of sports. To understand ExAdd’s etiology, longitudinal prospective studies are warranted, which consider the underlying motives for exercise participation in sports categories, such as resistance and fitness sports. Prevention and treatment strategies for excessive health behaviors should focus on helping women maintain a balanced approach to healthy eating and assisting men in regulating their exercise activities.

## Data Availability

The raw data supporting the conclusions of this article will be made available by the authors, without undue reservation.
