# Supplementary material for: Excessive health behaviors in sports: links of orthorexia nervosa and exercise addiction with well-being, exercise activity in sports categories, and gender effects
Source: Front Nutr. 2024 Dec 3;11:1494958. doi: 10.3389/fnut.2024.1494958 (PMC11649418; doi:10.3389/fnut.2024.1494958)
Supplement: Supplementary file 1 [file Table_1.pdf]

Wachten H, Wurst R, Paganini S and Strahler J (2024) Excessive health behaviors in sports: links of orthorexia nervosa and exercise addiction with well-being, exercise activity in sports categories, and gender effects.
Front. Nutr. 11:1494958.
doi: 10.3389/fnut.2024.1494958

Supplementary Table

Generalized *p*-values of parametric bootstrapping 2x2 ANOVAs with independent variables sport (no sports vs. indicated sports category), gender (women vs. men) and dependent variables Exercise Addiction, Orthorexia nervosa, Healthy Orthorexia and Well-Being.

	Endurance	Resistance and fitness	Health	Ball and team	Antigravitation	Technical	Aesthetic	Rehabilitation	Martial Arts
EAI-R									
Sport	**<.001**	**<.001**	**<.001**	**<.001**	**<.001**	**<.001**	**.006**	**.032**	**<.001**
Gender	.211	**<.001**	.921	.079	.976	.794	.889	.336	.963
SportxGender	.292	**.031**	.853	.071	.906	.506	.649	.219	.809
TOS OrNe									
Sport	.054	**.050**	.810	.242	.248	.471	.773	**.042**	**.031**
Gender	**.002**	**.003**	**.001**	**.012**	**<.001**	**.026**	**.049**	**<.001**	**.018**
SportxGender	.361	.438	.593	.566	.114	.223	.714	.088	.119
TOS HeOr									
Sport	**.001**	**<.001**	**<.001**	**.045**	**<.001**	.160	.147	.105	**.005**
Gender	.345	.371	.112	.327	.150	.389	**.044**	**.025**	.367
SportxGender	.362	.315	**.038**	.163	.737	.328	.088	.058	.368
WHO-5									
Sport	**<.001**	**<.001**	**<.001**	**<.001**	**<.001**	**<.001**	**.031**	.819	.180
Gender	**<.001**	**<.001**	.064	**.001**	**.015**	**.029**	.157	.081	**.022**
SportxGender	.752	.793	.409	.742	.879	.905	.474	.672	.668

Note. EAI-R = Exercise Addiction Inventory Revised; TOS HeOr = Healthy Orthorexia subscale of the Teurel Orthorexia Scale; TOS OrNe = Orthorexia nervosa subscale of the Teurel Orthorexia Scale; WHO-5 = WHO-5 Well Being Scale.
